# Extract

**Published:** 1875-04

**Authors:** 


*Extract.*

"I am glad you are making an earnest effort to give us a large and reliable Advertising department. It is important to have pure medicine. The safety of the sick and the reputation of the profession demands that we see that our home druggists deal with reliable houses North. If you will recommend them, we will call the attention of our druggists to the importance of dealing with druggists who will not deceive the profession—who warrant their drugs and chemicals to be all they are represented to be. * *

"Yours truly, G. C. B."

We have, since the establishment of this journal, acted upon the principles suggested above.

We are exerting ourselves to secure just that class of advertisers above-mentioned. We have already secured several and have the promise of others. We do not wish or solicit, knowingly, any firm as advertisers not up to the highest mark of respectability and reliability. The country is being flooded with worthless drugs, and the physician can never (except in rare instances) rely upon the fluid extracts, elixirs, etc. in the hands of our druggists. We desire to expose the deceptions and frauds practiced upon the doctors all over the land in this matter, and while we cannot reach the evil to its full extent, we can point our friends to those houses placing pure, and only pure, chemicals, drugs, elixirs, etc., upon the market.—We trust, in a very short time, to recommend to our readers a list of reliable houses the Southern trade can depend upon.

We say this for the benefit of the profession—for the good of our brethren and the people. The senior Editor is a member of the "State Board of Physicians to regulate the licensing of Physicians and Druggists in the State," and, as such, it becomes his duty to handle this subject in Georgia for the benefit of the people and the protection of the profession.

*Ayres' Hernia Truss.*

In this issue appears an advertisement of the above named hernial truss. We have not yet had an opportunity of trying this truss, but from the numerous recommendations of medical gentlemen, among whom we see the names of some of the most learned and distinguished of the profession in Virginia, we do not question its superior advantages over other mechanical appliances for the relief of hernia.

We shall make arrangements to have them kept on hand in Atlanta, where physicians and others within reach can procure them. Physicians or druggists outside of Atlanta can make arrangements to supply the demand for them in their localities, by addressing Samuel Ayres, Richmond, Virginia.

LIST OF CASH PAYMENTS SINCE FEBRUARY NUMBER.

| | | | |
|---|---|---|---|
| Dr. M. Giesy, Oregon | $3 00 | Dr. W. P. Anderson, Georgia | $3 00 |
| Dr. C. P. Sanders, Alabama | 3 00 | Dr. G. H. Thompson, Alabama | 3 00 |
| Dr. Louis Hadden, Louisiana | 3 00 | Dr. C. O. Nicholds, Arkansas | 50 |
| Dr. J. S. Bacon, Louisiana | 3 00 | Dr. J. E. Pope, Georgia | 3 00 |
| Dr. C. C. Hart, Georgia | 3 00 | Dr. S. T. Birdsong, Mississippi | 3 00 |
| Dr. L. W. Mobley, Georgia | 5 50 | Dr. G. W. Wimbish, Texas | 1 50 |
| Dr. A. A. Lyon, Mississippi | 3 00 | Dr. Z. T. Young, Louisiana | 3 00 |

